# Differences in neural connectivity between the substantia nigra and ventral tegmental area in the human brain

**DOI:** 10.3389/fnhum.2014.00041

**Published:** 2014-02-06

**Authors:** Hyeok Gyu Kwon, Sung Ho Jang

**Affiliations:** Department of Physical Medicine and Rehabilitation, College of Medicine, Yeungnam UniversityDaegu, South Korea

**Keywords:** diffusion tensor imaging, dopamine, substantia nigra, ventral tegmental area, structural connectivity

## Abstract

**Objectives:** Many animal and a few human studies have reported on the neural connectivity of the substantia nigra (SN) and the ventral tegmental area (VTA). However, it has not been clearly elucidated so far. We attempted to investigate any differences in neural connectivity of the SN/VTA in the human brain, using diffusion tensor imaging (DTI).

**Methods:** Sixty-three healthy subjects were recruited for this study. DTIs were acquired using a sensitivity-encoding head coil at 1. 5T. Connectivity was defined as the incidence of connection between the SN/VTA and each brain regions in the brain.

**Results:** The connectivity of SN was higher than that of the VTA. This included in the primary motor cortex, primary somatosensory cortex, premotor cortex, prefrontal cortex, caudate nucleus, globus pallidus, putamen, nucleus accumbens, temporal lobe, amygdala, pontine basis, occipital lobe, anterior and posterior lobe of cerebellum, corpus callosum, and external capsule (*p* < 0.05). However, no significant differences were observed in the red nucleus, thalamus, pontine tegmentum, and medial temporal lobe between the SN and VTA (*p* > 0.05).

**Conclusions:** We found the differences in neural connectivity of the SN/VTA in the human brain. The method and results of this study can provide useful information for clinicians and researchers in neuroscience, especially who work for Parkinson’s disease and patients with brain injury.

## Introduction

Dopamine is one of the major neurotransmitters in the brain and is a key regulator involved in motor skill learning, emotion, motivation, and cognition (Meyer and Quenzer, 2005; Mendoza and Foundas, 2007; Molina-Luna et al., 2009; Nestler et al., 2009; Kwak et al., 2010; Hosp et al., 2011; Kwak et al., 2012). There are four primary dopaminergic nuclei in the brain: the pars compacta of substantia nigra (SNc), the ventral tegmental area (VTA), the retrorubral area in the mesencephalic reticular formation, and the arcuate nucleus in the hypothalamus (Nestler et al., 2009). The SNc and VTA have been the targets for research of dopaminergic nuclei because the retrorubral area is relatively small comparing with SNc and VTA, and the arcuate nucleus is involved in prolatin secretion through the tuberoinfundibular pathway (Hirsch et al., 1992; Francois et al., 1999; Meyer and Quenzer, 2005; Düzel et al., 2009; Siegel and Sapru, 2010). In addition, the exact identification of the retrorubral area and arcuate nucleus using brain MRI is difficult in the live human brain.

The four major dopaminergic systems serve distinct functions, which include the nigrostriatal pathway, the mesolimbic pathway, the mesocortical pathway, and the tuberoinfundibular pathway (Meyer and Quenzer, 2005; Nestler et al., 2009). Previous animal studies have reported that the substantia nigra mainly works for the nigrostriatal pathway and the VTA for the mesolimbic and mesocortical pathways. Therefore, the nigrostriatal pathway is involved in voluntary movement, in contrast, the mesolimbic and mesocortical pathways are involved in cognition and emotion (Phillipson, 1979; Swanson, 1982; Oades and Halliday, 1987; van Domburg and ten Donkelaar, 1991). These results suggest that there may be differences in neural connectivity between the SN and the VTA. Many animal studies have reported on the neural connectivity of the SN/VTA (Phillipson, 1979; Swanson, 1982; Oades and Halliday, 1987; van Domburg and ten Donkelaar, 1991). A few studies have reported on this topic in the human brain, however, it has not been clearly elucidated so far (Düzel et al., 2009; Menke et al., 2010; Chowdhury et al., 2013).

Diffusion tensor imaging (DTI) has a unique advantage in evaluation of white matter by virtue of its ability to visualize water diffusion characteristics (Basser et al., 1994). Recently developed multi-tensor model DTI allows to estimate more than one fiber population in the each imaging voxel and suggests that probability corresponds to multiple fiber populations whereas the single tensor model DTI analyzes only a dominant fiber bundle (Smith et al., 2004; Parker and Alexander, 2005; Behrens et al., 2007). Many multi-tensor model DTI studies have reported on neural connectivity in normal subjects (Behrens et al., 2003b; Guye et al., 2003; Parker and Alexander, 2005; Jang et al., 2013). However, only few studies have reported on the difference of neural connectivity of the SN/VTA in the human brain (Menke et al., 2010; Chowdhury et al., 2013).

In the current study, we hypothesized that neural connectivity of the SN/VTA is different in the human brain and attempted to investigate differences in neural connectivity of the SN/VTA, using DTI.

## Methods

### Subjects

We recruited 63 healthy subjects (males: 31, females: 32, mean age: 37.9 years, range: 20–67 years) with no previous history of neurological, physical, or psychiatric illness for this study. All subjects understood the purpose of the study and provided written, informed consent prior to participation. The study protocol was approved by the Institutional Review Board of the Yeungnam university hospital (YUH-12-0421-O60).

### Data acquisition

DTI data were acquired using a 6-channel head coil on a 1.5 T Philips Gyroscan Intera (Philips, Ltd, Best, The Netherlands) with single-shot echo-planar imaging. For each of the 32 non-collinear diffusion sensitizing gradients, we acquired 67 contiguous slices parallel to the anterior commissure-posterior commissure line. Imaging parameters were as follows: acquisition matrix = 96 × 96; reconstructed to matrix = 128 × 128; field of view = 221 × 221 mm^2^; repetition time (TR) = 10,726 ms; echo time (TE) = 76 ms; parallel imaging reduction factor (SENSE factor) = 2; echo-planar imaging (EPI) factor = 49; *b* = 1000 s/mm^2^; number of excitations (NEX) = 1; and a slice thickness of 2.3 mm (acquired voxel size 1.73 × 1.73 × 2.3 mm^3^).

### Probabilistic fiber tracking

**Figure 1:**
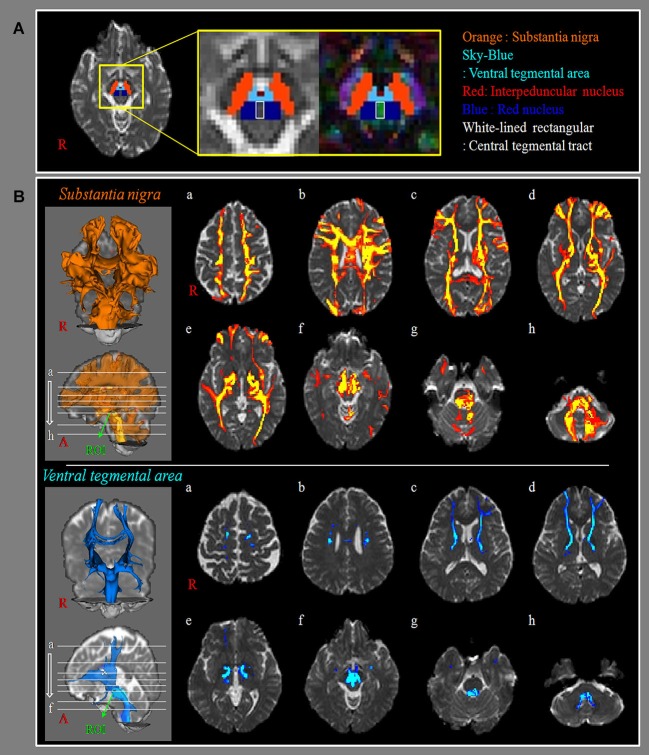
**Neural connectivity of substantia nigra and ventral tegmental area. (A)** The region of interest (ROI): a seed ROI for substantia nigra (SN, orange), is placed on the isolated SN of the upper midbrain on the B0 and color-coded map (dorsomedially next to the cerebral peduncle of the upper midbrain). A seed ROI for ventral tegmental area (VTA, sky-blue), is placed on the isolated VTA of the upper midbrain on the B0, and color-coded map. We use other structures to isolate the VTA such as interpeduncular nucleus (anterior boundary, red), central tegmental tract (posterior boundary, white-lined rectangular), midline (medial boundary), red nucleus (blue) and SN (lateral boundary). **(B)** SN/VTA: results of diffusion tensor tractography for the connectivity of SN/VTA (a: cortex level, b: upper corona radiata level, c: upper internal capsule level, d: lower internal capsule level, e: bicommissure level f: midbrain level, g: upper pons level, h: lower pons level). a to b levels: primary motor cortex, primary somatosensory cortex, premotor cortex, prefrontal cortex, corpus callosum, c to e levels: caudate nucleus, putamen, globus pallidus, nucleus accumbens, thalamus, external capsule, f to h level: red nucleus, amygdala, medial temporal lobe, temporal lobe pontine basis, pontine tegmentum, anterior lobe of cerebellum, posterior lobe of cerebellum.

The Oxford Centre for Functional Magnetic Resonance Imaging of the Brain (FMRIB) Software Library (FSL^1^) was used for analysis of diffusion-weighted imaging data. Affine multi-scale two-dimensional registration was used for correction of head motion effect and image distortion due to the eddy current. Mean translation and rotation was observed the sub-one pixel (0.51 ± 0.47 mm). Fiber tracking was performed using a probabilistic tractography method based on a multi-fiber model, and applied in the present study utilizing tractography routines implemented in FMRIB Diffusion (5000 streamline samples, 0.5 mm step lengths, curvature thresholds = 0.2) (Behrens et al., 2003a; Smith et al., 2004; Behrens et al., 2007). This fiber tracking method by multi-fiber model calculated and generated 5000 streamline samples from seed region of interest (ROI) with consideration of the both dominant and non-dominant orientation of diffusion in each voxel and showed how connects the brain regions. Therefore, it has advantage to solve the problem of the crossing fiber. Especially, cross points of the corpus callosum and corona radiata, corticospinal tract fibers and pontocerebellar fibers at pons, and superior and medial frontal gyri are known to be the crossing fiber point (Wiegell et al., 2000). For the connectivity of the SN, a seed ROI was placed on the isolated SN of the upper midbrain on the color-coded map (dorsomedially next to the cerebral peduncle of the upper midbrain) (Mori et al., 2004). For the connectivity of the VTA, a seed ROI was placed on the VTA in the upper midbrain on the color-coded map. We identified the VTA by reconstructing the adjacent structures: interpeduncular nucleus (anterior boundary), central tegmental tract (posterior (www.fmrib.ox.ac.uk/fsl) boundary), midline (medial boundary), red nucleus and SN (lateral boundary) (Mori et al., 2004; Habas and Cabanis, 2007; Blood et al., 2010; Figure 1A). Out of 5000 samples generated from the seed voxel, results for contact were visualized with the threshold at a minimum of five streamline through each voxel for analysis. Connectivity represented the percentage as all hemispheres of 63 subjects. On the other hand, we measured the size of ROI for the SN and VTA.

### Determination of connection between substantia nigra (SN), ventral tegmental area (VTA) and brain regions

Connectivity was defined as the incidence of connection between the SN/VTA and each brain region: primary motor cortex (M1, brodmann area [BA]: 4), primary somatosensory cortex (S1, BA: 1, 2, 3), premotor cortex (PMC, BA: 6), prefrontal cortex (BA: 9, 10, 11, 12), caudate nucleus, putamen, globus pallidus, nucleus accumbens, thalamus, external capsule, red nucleus, amygdala, medial temporal lobe (BA: 27, 28, 34, 35, 36, 37), temporal lobe (superior, middle, inferior, BA: 20, 21, 22), pontine basis, pontine tegmentum, anterior lobe of cerebellum, posterior lobe of cerebellum, corpus callosum, and occipital lobe (BA: 17, 18, 19).

### Statistical analysis

SPSS software (v.15.0; SPSS, Chicago, IL) was used for statistical analysis. The Chi-square test was used for determination of the difference in connectivity between the right and left hemispheres, and between the SN and VTA. In addition, we performed an independent *t*-test for determination of differences in size of ROI between the SN and VTA, and between the right and left hemispheres. The significant level of the *p* value was set at 0.05.

## Results

Connectivity of the SN/VTA is summarized in Table 1. In all subjects, the SN showed 100% connectivity to the red nucleus, thalamus, globus pallidus, corpus callosum, M1, S1, PMC, pontine tegmentum, and posterior lobe of cerebellum. By contrast, other brain regions showed over 70% connectivity: caudate nucleus, putamen, nucleus accumbens, prefrontal cortex, temporal lobe, amygdala, pontine basis, occipital lobe, and anterior lobe of cerebellum. As for connectivity of the VTA, red nucleus, thalamus, and pontine tegmentum revealed 100% connectivity in all subjects. By contrast, the VTA revealed over 70% connectivity in the following brain regions: globus pallidus, posterior lobe of cerebellum, prefrontal cortex, M1, and amygdala (Figure 1B). On the other hand, the sizes of ROI for the SN and VTA were 40.3 ± 5.2 mm and 10.8 ± 1.9 mm, respectively.

**Table 1:** **Comparison of connectivity between substantia nigra and ventral tegmental area with brain regions**.

Brain regions	SN	VTA	*p*
Red nucleus	100%	100%	1
Thalamus	100%	100%	1
Globus pallidus	100%	93.65%	.007*
Corpus callosum	100%	28.57%	.000*
Primary motor cortex (BA: 4)	100%	70.63%	.000*
Primary somatosensory cortex (BA: 1, 2, 3)	100%	36.51%	.000*
Premotor cortex (BA: 6)	100%	29.37%	.000*
Pontine tegmentum	100%	100%	1
Posterior lobe of cerebellum	100%	89.68%	.000*
Caudate nucleus	99.21%	68.25%	.000*
Putamen	99.21%	68.25%	.000*
Nucleus accumbens	97.62%	65.87%	.000*
Prefrontal cortex (BA: 9, 10, 11, 12)	95.24%	73.81%	.000*
Temporal lobe (BA: 20, 21, 22)	88.89%	63.49%	.000*
Amygdala	84.92%	70.63%	.006*
Pontine basis	80.95%	8.73%	.000*
Occipital lobe (BA: 17, 18, 19)	76.98%	34.13%	.000*
Anterior lobe of cerebellum	70.63%	2.38%	.000*
External capsule	50.79%	0	.000*
Medial temporal lobe (BA: 27, 28, 34, 35, 36, 37)	13.49%	6.35%	.058

SN Substantia nigra VTA Ventral tegmental area BA brodmann area

*p*: statistics value for comparison to the connectivity between SN and VTA, Connectivity (%),* : *p* < 0.05

In comparison to the difference of connectivity between the SN and the VTA, there were significant differences in the globus pallidus (*p* = 0.007), corpus callosum (*p* = 0.000), M1 (*p* = 0.000), S1 (*p* = 0.000), PMC (*p* = 0.000), posterior lobe of cerebellum (*p* = 0.000), caudate nucleus (*p* = 0.000), putamen (*p* = 0.000), nucleus accumbens (*p* = 0.000), prefrontal cortex (*p* = 0.000), temporal lobe (*p* = 0.000), amygdala (*p* = 0.006), pontine basis (*p* = 0.000), occipital lobe (*p* = 0.000), anterior lobe of cerebellum (*p* = 0.000), and external capsule (*p* = 0.000) (*p* < 0.05). However, no significant differences were observed in the red nucleus (*p* = 1.000), thalamus (*p* = 1.000), pontine tegmentum (*p* = 1.000), and medial temporal lobe (*p* = 0.058) between the SN and VTA (*p* > 0.05) In addition, there were no significant differences in connectivity between right and left hemispheres (*p* > 0.05). Regarding the size of ROI, significant difference was observed in size of ROI between the SN and VTA (*p* < 0.05), however, no significant difference was observed in size of ROI for the SN and VTA between the right and left hemispheres (*p* > 0.05).

## Discussion

In the current study, using multi-tensor model DTI, we investigated differences in neural connectivity between the SN and the VTA. We observed that: (1) the SN showed more than 70% connectivity in all brain regions except for external capsule and medial temporal lobe, whereas the VTA showed less than 70% connectivity in many brain regions (corpus callosum, S1, PMC, caudate nucleus, putamen, nucleus accumbens, temporal-occipital lobe, pontine basis, anterior lobe of cerebellum, external capsule); (2) in several specific brain areas, the connectivity of the SN was higher than in the VTA: basal ganglia, primary sensori-motor cortex, PMC, prefrontal cortex, nucleus accumbens, cerebellum, corpus callosum, temporooccipital lobe, amygdala, and pontine basis.

Previous studies have reported that about 75% of dopamine neurons existed in the SNc, 15% in the VTA, and 10% in the retrorubral field in human and primates (Hirsch et al., 1992; Francois et al., 1999; Düzel et al., 2009). These data indicate that the neural connectivity of the SN could be much higher than that of the VTA. Therefore, our results that the neural connectivity of the SN was higher than that of the VTA are in accordance with the results of previous studies (Hirsch et al., 1992; Francois et al., 1999; Düzel et al., 2009). As for the connection areas, many animal studies have described the difference in the working areas of the SN and VTA (Phillipson, 1979; Swanson, 1982; Oades and Halliday, 1987; van Domburg and ten Donkelaar, 1991). The classical concept was that the SNc mainly works for the nigrostriatal pathway, whereas the VTA works for both the mesolimbic and mesocortical pathways (Phillipson, 1979; Swanson, 1982; Oades and Halliday, 1987; van Domburg and ten Donkelaar, 1991). Therefore, it has been known that SNc is mainly connected with the striatum and VTA is mainly with nucleus accumbens and frontal cortex. However, in 2009, Düzel et al. described that the functional difference between the SNc and the VTA seemed subtle (Düzel et al., 2009). Furthermore, they reported that the mesolimbic and mesocortical dopaminergic system were dispersed throughout the SN/VTA in the human brain (Düzel et al., 2009). Our results that the whole SN showed more neural connectivity to the frontal cortex and nucleus accumbens as well as basal ganglia compared to the VTA appears to be coincided with the Düzel’s study.

To the best of our knowledge, only few studies have reported on the neural connectivity of the SN or the VTA in the human brain, using DTI (Menke et al., 2010; Chowdhury et al., 2013). In 2011, Menke et al. divided the SN into the SNc and the pars reticularis of the SN (SNr) using segmentation (Menke et al., 2010). These maps showed that the SNc was connected with the posterior striatum, the pallidum, the anterior limb of the internal capsule, anterior thalamic nuclei, and anterior thalamic radiation leading to the prefrontal cortex. By contrast, the SNr was connected to the posterior striatum, ventral thalamus, posterior limb of internal capsule, and tracts leading to premotor and primary sensori-motor cortices. The fact that we could not isolate the SNc from the SNr is a limitation of this study. That was because we could not identify the SNc from the SN on conventional MRI and DTI. Recently, Chowdhury et al. (2013) investigated the connectivity between the SN and the striatum by parcellation of the SN based on the anatomical connectivity to the striatum (Chowdhury et al., 2013). They found that a dorsomedial region of the SN preferentially connected to the ventral striatum (nucleus accumbens) whereas a more ventrolateral region of the SN connected to the dorsal striatum (the caudate and putamen). In addition, the connectivity of the dorsomedial region of the SN to the ventral striatum showed a positive correlation with reward dependence score. Lack of behavioral data in this study would be a limitation of this study. Consequently, this is the first DTI study that compares differences in neural connectivity of the VTA/SN in the human brain.

In conclusion, we found that the SN showed more neural connectivity to fronto-temporo-occipital lobe, cerebellum, and nucleus accumbens as well as basal ganglia compared to the VTA. The method and results of this study can provide useful information for clinicians and researchers in neuroscience, especially who work for Parkinson’s disease and patients with brain injury. However, several limitations of DTI should be considered (Lee et al., 2005; Parker and Alexander, 2005; Yamada, 2009; Fillard et al., 2011; Jeurissen et al., 2013). First, DTI may underestimate the fiber tracts. DTI is a powerful anatomic imaging tool that can demonstrate gross fiber architecture, but not functional or synaptic connections. However, the probabilistic approach which was adopted in this study can lead to less serious underestimation because this approach would depict more fibers than the deterministic approach (Yamada et al., 2009). Second, DTI could lead to both false positive and negative throughout the white matter of brain because of complex fiber configurations such as crossing or kissing fiber and partial volume effects (Lee et al., 2005; Parker and Alexander, 2005; Yamada, 2009; Fillard et al., 2011; Jeurissen et al., 2013). Third, the low tesla (1.5), channels (6), and diffusion directions (32) which were employed in this study are another limitations of this study. We suggest further DTI studies to overcome these DTI limitations which may not estimate the real neural connectivity accurately.

## Conflict of interest statement

The authors declare that the research was conducted in the absence of any commercial or financial relationships that could be construed as a potential conflict of interest.

## Footnotes

^1^www.fmrib.ox.ac.uk/fsl
